# Supplementary material for: A Systematic Investigation of Lipid Transfer Proteins Involved in Male Fertility and Other Biological Processes in Maize
Source: Int J Mol Sci. 2023 Jan 14;24(2):1660. doi: 10.3390/ijms24021660 (PMC9864150; doi:10.3390/ijms24021660)
Supplement: Supplementary file 1 [file ijms-24-01660-s001.zip › ijms-2096457-supplementary.pdf]

Supplementary Materials:

Article

A Systematic Investigation of Lipid Transfer Proteins Involved in Male Fertility and Other Biological Processes in Maize

Chaowei Fang †, Suowei Wu †, Ziwen Li, Shuangshuang Pan, Yuru Wu, Xueli An, Yan Long, Xun Wei * and Xiangyuan Wan *

Zhongzhi International Institute of Agricultural Biosciences, Research Center of Biology and Agriculture, School of Chemistry and Biological Engineering, University of Science and Technology Beijing (USTB), Beijing 100096, China

* Correspondence: weixun@ustb.edu.cn (X.W.); wanxiangyuan@ustb.edu.cn (X.W.); Tel.: +86-(189)-1087-6260 (X.W.); +86-(186)-0056-1850 (X.W.)

† These authors contributed equally to this work.

Table S1. Information of 65 maize *LTP* genes.

Name	Gene ID (B73 V3)	Gene ID (B73 V4)	Synonyms in MaizeGDB	Chr.	Physical locus in B73 genome V4	Physical locus in B73 genome V5	Length (aa)	Theoretical pI	Predicted subcellular localization
**Type 1**									
ZmLTP1.1	GRMZM2G126397	Zm00001d043049	plt3	3	187418004..187422004	189563998..189568057	123	9.38	Cell membrane, Cell wall
ZmLTP1.2-1	GRMZM2G010868	Zm00001d044686	plt2	3	234928537..234931896	236775414..236779439	123	9.38	Cytoplasm
ZmLTP1.2-2						236775430..236779315	125	7.17	Cell membrane, Cell wall
ZmLTP1.3-1	GRMZM2G107839	Zm00001d044685	-	3	234863389..234868848	236712251..236716177	121	9.27	Cell wall
ZmLTP1.3-2						236712397..236716333	123	9.54	Nucleus
ZmLTP1.4	GRMZM2G096234	Zm00001d008950	-	8	26734011..26737786	26586162..26590124	170	11.22	Cell wall
ZmLTP1.5	GRMZM2G025026	Zm00001d046158	-	9	68299557..68302910	70375078..70379060	126	6.01	Cell wall
ZmLTP1.6	GRMZM2G101958	Zm00001d023343	plt1	10	3823249..3827245	3757633..3761629	119	4.69	Cell membrane, Cell wall
ZmLTP1.7	GRMZM5G898755	Zm00001d023344	-	10	3838740..3856748	3786912..3791169	118	9.08	Cell membrane
ZmLTP1.8-1	GRMZM2G439268	Zm00001d024710	-	10	84545407..84555133	86312524..86323137	175	8.78	Cell wall
ZmLTP1.8-2						86312892..86322593	135	8.40	Nucleus
**Type 2**									
ZmLTP2.1	GRMZM2G137329	Zm00001d027332	-	1	2991511..2995234	3084661..3088276	98	9.25	Cell membrane
ZmLTP2.2	GRMZM2G081464	Zm00001d007299	-	2	227229498..227232995	227203121..227206602	98	9.25	Cell membrane
ZmLTP2.3	GRMZM2G406552	Zm00001d052759	-	4	199956827..199960120	201803358..201806936	99	9.12	Cell membrane
ZmLTP2.4	GRMZM2G387360	Zm00001d013907	-	5	24371450..24374740	24780624..24784315	97	8.86	Cell membrane
ZmLTP2.5	GRMZM2G039383	Zm00001d036452		6	89056590..89059874	95606693..95610394	96	8.74	Cell membrane
ZmLTP2.6	GRMZM2G403007	Zm00001d037677		6	133753395..133756700	140571718..140575248	94	9.01	Cell membrane
ZmLTP2.7	GRMZM2G004909	Zm00001d038911		6	166850024..166853329	174166937..174170679	101	8.98	Cell membrane
ZmLTP2.8	GRMZM2G320373	Zm00001d038913	ltp1	6	166866833..166870129	174183772..174187444	101	8.83	Cell membrane
ZmLTP2.9	GRMZM2G393150	Zm00001d011630		8	156785611..156788913	156074621..156078392	98	9.65	Cell membrane
**Type C**									
ZmLTPc1	AC225127.3_FG003	Zm00001d052736	ms44	4	199342499..199346167	201189284..201192696	103	7.52	Cell membrane
ZmLTPc2	GRMZM2G073377	Zm00001d021226	nthr3	7	146555258..146559128	147778434..147782302	103	7.52	Cell membrane
**Type D**									

ZmLTPd1	GRMZM2G031102	-	-	1	-	-	111.00	8.49	Cell membrane
ZmLTPd2	GRMZM2G071771	-	-	2	-	-	156	8.50	Cell membrane
ZmLTPd3	GRMZM2G136364	Zm00001d003757	-	2	57739376..57743342	57628036..57631949	111	8.49	Cell membrane
ZmLTPd4	GRMZM2G099867	Zm00001d004700	-	2	132136657..132140680	132045819..132049539	115	6.05	Cell membrane
ZmLTPd5	GRMZM2G164440	Zm00001d005146	-	2	161021720..161025067	160904727..160908766	107	8.74	Cell membrane
ZmLTPd6	GRMZM2G087413	Zm00001d041822	betl9	3	139333675..139337330	140244639..140248303	106	8.77	Cell membrane, Nucleus
ZmLTPd7	GRMZM2G396418	Zm00001d042383	-	3	164036835..164040155	164558045..164561365	118	9.30	Cell membrane
ZmLTPd8	GRMZM2G065557	Zm00001d042862	-	3	182289284..182293070	184483458..184487254	116	6.68	Cell membrane
ZmLTPd9	GRMZM2G094632	Zm00001d014315	-	5	40640614..40644780	40904570..40908818	103	8.72	Cell membrane
ZmLTPd10	AC233926.1_FG002	Zm00001d019399	-	7	32079614..32082925	32079614..32082925	105	6.68	Cell membrane
ZmLTPd11	GRMZM2G000221	Zm00001d019400	-	7	32220531..32223848	31964542..31968231	107	8.35	Cell membrane
ZmLTPd12	GRMZM2G471051	Zm00001d012080	-	8	167760881..167764189	169059891..169063740	100	8.91	Cell membrane
ZmLTPd13	GRMZM2G170969	Zm00001d012081	-	8	167764716..167768018	169063941..169067513	111	7.49	Cell membrane, Cell wall
ZmLTPd14-1	GRMZM2G091054	Zm00001d012572	al9	8	176807113..176814509	178062064..178065852	117	6.00	Cell membrane, Chloroplast, Nucleus
ZmLTPd14-2						178066088..178069542	91	8.42	Cell membrane
ZmLTPd15	GRMZM2G155555	Zm00001d024958	-	10	96866696..96870584	98671565..98675351	120	8.93	Cell membrane
ZmLTPd16	GRMZM2G063375	Zm00001d024959	-	10	96879402..96883182	98684217..98687993	120	8.93	Cell membrane
**Type G**									
ZmLTPg1-1	GRMZM5G850455	Zm00001d027811	-	1	14309986..14319956	14212854..14217190	189	7.46	Cell membrane
ZmLTPg1-2		Zm00001d027812	-				181	8.32	Cell membrane
ZmLTPg2	GRMZM2G174680	Zm00001d028973	plt4	1	53307564..53311100	52766145..52770357	181	8.01	Cell membrane
ZmLTPg3	GRMZM2G078876	Zm00001d029365	-	1	66856799..66862704	66856799..66862704	161	8.49	Cell membrane
ZmLTPg4	GRMZM2G083725	Zm00001d031636	ltpg1	1	197044858..197049701	199011874..199016801	182	8.89	Cell membrane
ZmLTPg5	GRMZM2G097137	Zm00001d033595	-	1	268071263..268075125	269848021..269851957	232	10.08	Cell membrane
ZmLTPg6	GRMZM2G006047	Zm00001d034414	-	1	292294256..292298377	293918516..293922882	216	6.17	Cell membrane
ZmLTPg7	GRMZM2G005991	Zm00001d034415	plt5	1	292329708..292333982	293953981..293958346	202	8.07	Cell membrane
ZmLTPg8	GRMZM2G065640	Zm00001d034416	-	1	292374878..292378806	293999142..294003829	278	9.44	Cell membrane
ZmLTPg9	GRMZM2G168833	Zm00001d034513	-	1	295270975..295276495	296821940..296827044	260	8.00	Cell membrane

ZmLTPg10	GRMZM2G414620	Zm00001d003405	-	2	43285551..43289511	43273688..43277906	160	8.81	Cell membrane
ZmLTPg11	GRMZM2G151021	Zm00001d003600	-	2	49390793..49396018	49302556..49307786	195	8.53	Cell membrane
ZmLTPg12	AC194203.3_FG002	Zm00001d006915	-	2	219301495..219304917	219280230..219284712	232	9.30	Cell membrane
ZmLTPg13	GRMZM2G176347	Zm00001d007832	-	2	240515422..240519824	239843482..239847829	169	7.12	Cell membrane
ZmLTPg14	GRMZM2G071575	Zm00001d049834	-	4	47100636..47104875	47397812..47402451	187	6.79	Cell membrane
ZmLTPg15	GRMZM2G145054	Zm00001d050904	-	4	129473894..129477703	131387877..131391825	140	8.14	Cell membrane
ZmLTPg16	GRMZM2G130454	Zm00001d013089	-	5	4533909..4538027	4714722..4718941	258	9.28	Cell membrane
ZmLTPg17	GRMZM2G141858	Zm00001d014513	-	5	51112698..51118295	51229578..51235168	149	8.46	Cell membrane
ZmLTPg18	GRMZM2G116167	Zm00001d014758	plt6	5	61806171..61810517	61837211..61841796	256	10.21	Cell membrane
ZmLTPg19	GRMZM2G089400	Zm00001d036550	-	6	91655493..91658945	98774564..98779902	220	7.50	Cell membrane
ZmLTPg20	GRMZM2G089288	Zm00001d018915	-	7	9191174..9195318	9073465..9079182	206	8.67	Nucleus
ZmLTPg21	GRMZM2G046750	Zm00001d018926	-	7	9490940..9495219	9373449..9377734	169	9.28	Cell membrane
ZmLTPg22	GRMZM2G170044	Zm00001d019100	-	7	16650048..16655882	16546343..16552199	194	5.53	Cell membrane
ZmLTPg23	GRMZM2G171597	Zm00001d022195	-	7	172626913..172631128	176124670..176129139	203	8.72	Cell membrane
ZmLTPg24	GRMZM2G379035	Zm00001d012535	-	8	176257316..176261570	177512287..177516630	234	5.88	Cell membrane
ZmLTPg25	GRMZM2G004466	Zm00001d048202	-	9	152374510..152378805	155697596..155702084	203	8.72	Cell membrane
ZmLTPg26	GRMZM2G140646	Zm00001d023683	-	10	15303069..15309058	15100610..15106666	169	6.04	Cell membrane
**Single**									
ZmLTPx1	GRMZM2G036063	Zm00001d019628	-	7	46877998..46882274	46480634..46484818	169	6.04	Cell membrane
ZmLTPx2	GRMZM2G166484	Zm00001d025467	-	10	119823627..119828159	121410873..121416011	208	6.48	Cell membrane

Notes: 1. Information of maize *LTP* genes was acquired from MaizeGDB (https://maizegdb.org/). 2. Theoretical pI was retrieved in https://web.expasy.org/protparam/. 3. The prediction of subcellular localization information was retrieved in http://www.csbio.sjtu.edu.cn/bioinf/Cell-PLoc-2/.

Table S2. GO analysis of 65 *ZmLTP* genes

Class	ID	Description	Pvalue	Qvalue	Fg_num	Bg_num	Ratio	Genes
Biological Process	GO:0006869	lipid transport	2.89E-21	1.18E-19	11	128	0.086	*ZmLTPg8, ZmLTPg12, ZmLTP1.3-1, ZmLTP1.2-2, ZmLTP2.6, ZmLTP2.7, ZmLTP2.8, ZmLTP2.9, ZmLTPg25, ZmLTP1.7, ZmLTP1.8-1*
Biological Process	GO:0010876	lipid localization	6.80E-21	1.39E-19	11	138	0.08	*ZmLTPg8, ZmLTPg12, ZmLTP1.3-1, ZmLTP1.2-2, ZmLTP2.6, ZmLTP2.7, ZmLTP2.8, ZmLTP2.9, ZmLTPg25, ZmLTP1.7, ZmLTP1.8-1*
Biological Process	GO:0009627	systemic acquired resistance	1.66E-11	2.26E-10	5	28	0.179	*ZmLTPd3, ZmLTPd5, ZmLTPd7, ZmLTPd10, ZmLTPd11*
Biological Process	GO:0009814	defense response, incompatible interaction	6.33E-11	6.48E-10	5	36	0.139	*ZmLTPd3, ZmLTPd5, ZmLTPd7, ZmLTPd10, ZmLTPd11*
Biological Process	GO:0033036	macromolecule localization	5.54E-10	4.55E-09	11	1349	0.008	*ZmLTPg8, ZmLTPg12, ZmLTP1.3-1, ZmLTP1.2-2, ZmLTP2.6, ZmLTP2.7, ZmLTP2.8, ZmLTP2.9, ZmLTPg25, ZmLTP1.7, ZmLTP1.8-1*
Biological Process	GO:0071702	organic substance transport	3.00E-09	1.78E-08	11	1581	0.007	*ZmLTPg8, ZmLTPg12, ZmLTP1.3-1, ZmLTP1.2-2, ZmLTP2.6, ZmLTP2.7, ZmLTP2.8, ZmLTP2.9, ZmLTPg25, ZmLTP1.7, ZmLTP1.8-1*
Biological Process	GO:0006955	immune response	3.47E-09	1.78E-08	5	78	0.064	*ZmLTPd3, ZmLTPd5, ZmLTPd7, ZmLTPd10, ZmLTPd11*
Biological Process	GO:0045087	innate immune response	3.47E-09	1.78E-08	5	78	0.064	*ZmLTPd3, ZmLTPd5, ZmLTPd7, ZmLTPd10, ZmLTPd11*
Biological Process	GO:0002376	immune system process	5.71E-09	2.60E-08	5	86	0.058	*ZmLTPd3, ZmLTPd5, ZmLTPd7, ZmLTPd10, ZmLTPd11*
Biological Process	GO:0098542	defense response to other organism	8.74E-07	3.58E-06	5	235	0.021	*ZmLTPd3, ZmLTPd5, ZmLTPd7, ZmLTPd10, ZmLTPd11*
Biological Process	GO:0044765	single-organism transport	1.07E-06	3.99E-06	11	2779	0.004	*ZmLTPg8, ZmLTPg12, ZmLTP1.3-1, ZmLTP1.2-2, ZmLTP2.6, ZmLTP2.7, ZmLTP2.8, ZmLTP2.9, ZmLTPg25, ZmLTP1.7, ZmLTP1.8-1*
Biological Process	GO:1902578	single-organism localization	1.38E-06	4.05E-06	11	2848	0.004	*ZmLTPg8, ZmLTPg12, ZmLTP1.3-1, ZmLTP1.2-2, ZmLTP2.6, ZmLTP2.7, ZmLTP2.8, ZmLTP2.9, ZmLTPg25, ZmLTP1.7, ZmLTP1.8-1*
Biological Process	GO:0043207	response to external biotic stimulus	1.38E-06	4.05E-06	5	258	0.019	*ZmLTPd3, ZmLTPd5, ZmLTPd7, ZmLTPd10, ZmLTPd11*
Biological Process	GO:0051707	response to other organism	1.38E-06	4.05E-06	5	258	0.019	*ZmLTPd3, ZmLTPd5, ZmLTPd7, ZmLTPd10, ZmLTPd11*
Biological Process	GO:0009607	response to biotic stimulus	2.54E-06	6.95E-06	5	292	0.017	*ZmLTPd3, ZmLTPd5, ZmLTPd7, ZmLTPd10, ZmLTPd11*

Biological Process	GO:0006810	transport	5.85E-06	1.50E-05	11	3286	0.003	*ZmLTPg8, ZmLTPg12, ZmLTP1.3-1, ZmLTP1.2-2, ZmLTP2.6, ZmLTP2.7, ZmLTP2.8, ZmLTP2.9, ZmLTPg25, ZmLTP1.7, ZmLTP1.8-1*
Biological Process	GO:0051234	establishment of localization	6.76E-06	1.63E-05	11	3334	0.003	*ZmLTPg8, ZmLTPg12, ZmLTP1.3-1, ZmLTP1.2-2, ZmLTP2.6, ZmLTP2.7, ZmLTP2.8, ZmLTP2.9, ZmLTPg25, ZmLTP1.7, ZmLTP1.8-1*
Biological Process	GO:0051179	localization	9.52E-06	2.17E-05	11	3450	0.003	*ZmLTPg8, ZmLTPg12, ZmLTP1.3-1, ZmLTP1.2-2, ZmLTP2.6, ZmLTP2.7, ZmLTP2.8, ZmLTP2.9, ZmLTPg25, ZmLTP1.7, ZmLTP1.8-1*
Biological Process	GO:0009605	response to external stimulus	1.44E-05	3.10E-05	5	417	0.012	*ZmLTPd3, ZmLTPd5, ZmLTPd7, ZmLTPd10, ZmLTPd11*
Biological [illegible]	GO:0051704	multi-organism process	2.03E-05	4.16E-05	5	448	0.011	*ZmLTPd3, ZmLTPd5, ZmLTPd7, ZmLTPd10, ZmLTPd11*
Biological Process	GO:0006952	defense response	4.18E-05	8.17E-05	5	521	0.01	*ZmLTPd3, ZmLTPd5, ZmLTPd7, ZmLTPd10, ZmLTPd11*
Biological Process	GO:0006950	response to stress	0.017361	0.032354	5	2003	0.002	*ZmLTPd3, ZmLTPd5, ZmLTPd7, ZmLTPd10, ZmLTPd11*
Biological Process	GO:0050896	response to stimulus	0.148288	0.26434	5	3615	0.001	*ZmLTPd3, ZmLTPd5, ZmLTPd7, ZmLTPd10, ZmLTPd11*
Biological Process	GO:0044699	single-organism process	0.245005	0.418549	11	11354	0.001	*ZmLTPg8, ZmLTPg12, ZmLTP1.3-1, ZmLTP1.2-2, ZmLTP2.6, ZmLTP2.7, ZmLTP2.8, ZmLTP2.9, ZmLTPg25, ZmLTP1.7, ZmLTP1.8-1*
Biological Process	GO:0006468	protein phosphorylation	0.801839	1	1	1932	0.001	*ZmLTP1.8-2*
Biological Process	GO:0016310	phosphorylation	0.903279	1	1	2731	0	*ZmLTP1.8-2*
Biological Process	GO:0006796	phosphate-containing compound metabolic	0.964273	1	1	3786	0	*ZmLTP1.8-2*
Biological Process	GO:0006793	phosphorus metabolic process	0.965436	1	1	3820	0	*ZmLTP1.8-2*
Biological Process	GO:0006464	cellular protein modification process	0.974413	1	1	4126	0	*ZmLTP1.8-2*
Biological Process	GO:0036211	protein modification process	0.974413	1	1	4126	0	*ZmLTP1.8-2*
Biological Process	GO:0043412	macromolecule modification	0.981425	1	1	4446	0	*ZmLTP1.8-2*
Biological Process	GO:0044267	cellular protein metabolic process	0.99525	1	1	5743	0	*ZmLTP1.8-2*
Biological Process	GO:0019538	protein metabolic process	0.997895	1	1	6469	0	*ZmLTP1.8-2*
Biological Process	GO:0044260	cellular macromolecule metabolic process	0.999989	1	1	10379	0	*ZmLTP1.8-2*

Biological Process	GO:0043170	macromolecule metabolic process	0.999997	1	1	11287	0	*ZmLTP1.8-2*
Biological Process	GO:0044237	cellular metabolic process	1	1	1	13653	0	*ZmLTP1.8-2*
Biological Process	GO:0044238	primary metabolic process	1	1	1	13664	0	*ZmLTP1.8-2*
Biological Process	GO:0071704	organic substance metabolic process	1	1	1	14297	0	*ZmLTP1.8-2*
Biological Process	GO:0008152	metabolic process	1	1	1	16449	0	*ZmLTP1.8-2*
Biological Process	GO:0009987	cellular process	1	1	1	17455	0	*ZmLTP1.8-2*
Biological Process	GO:0008150	biological_process	1	1	17	21280	0.001	*ZmLTPg8, ZmLTPd3, ZmLTPd5, ZmLTPg12, ZmLTPd7, ZmLTP1.3-1, ZmLTP1.2-2, ZmLTP2.6, ZmLTP2.7, ZmLTP2.8, ZmLTPd10, ZmLTPd11, ZmLTP2.9, ZmLTPg25, ZmLTP1.7, ZmLTP1.8-1, ZmLTP1.8-2*
Cellular Component	GO:0016020	membrane	0.016842	0.101052	8	9885	0.001	*ZmLTPg2, ZmLTPg10, ZmLTPg13, ZmLTP1.3-1, ZmLTP1.2-2, ZmLTPg24, ZmLTP1.8-1, ZmLTP1.8-2*
Cellular Component	GO:0016021	integral component of membrane	0.267895	0.403415	5	8084	0.001	*ZmLTPg2, ZmLTPg10, ZmLTPg13, ZmLTPg24, ZmLTP1.8-2*
Cellular Component	GO:0031224	intrinsic component of membrane	0.280264	0.403415	5	8202	0.001	*ZmLTPg2, ZmLTPg10, ZmLTPg13, ZmLTPg24, ZmLTP1.8-2*
Cellular Component	GO:0005576	extracellular region	0.291233	0.403415	1	757	0.001	*ZmLTPc2(nthr3)*
Cellular Component	GO:0044425	membrane part	0.336179	0.403415	5	8712	0.001	*ZmLTPg2, ZmLTPg10, ZmLTPg13, ZmLTPg24, ZmLTP1.8-2*
Cellular Component	GO:0005575	cellular_component	1	1	9	20177	0	*ZmLTPg2, ZmLTPg10, ZmLTPg13, ZmLTP1.3-1, ZmLTP1.2-2, ZmLTPc2(nthr3), ZmLTPg24, ZmLTP1.8-1, ZmLTP1.8-2*
Molecular Function	GO:0008289	lipid binding	1.27E-22	4.20E-21	12	293	0.041	*ZmLTPg8, ZmLTPd3, ZmLTPd5, ZmLTPg12, ZmLTPd7, ZmLTP1.3-1, ZmLTP1.2-2, ZmLTPd10, ZmLTPd11, ZmLTPg25, ZmLTP1.7, ZmLTP1.8-1*
Molecular Function	GO:0005504	fatty acid binding	2.89E-12	4.77E-11	5	30	0.167	*ZmLTPd3, ZmLTPd5, ZmLTPd7, ZmLTPd10, ZmLTPd11*
Molecular Function	GO:0033293	monocarboxylic acid binding	5.24E-11	5.76E-10	5	52	0.096	ZmLTPd3, ZmLTPd5, ZmLTPd7, ZmLTPd10, ZmLTPd11
Molecular Function	GO:0031406	carboxylic acid binding	2.32E-09	1.53E-08	5	109	0.046	ZmLTPd3, ZmLTPd5, ZmLTPd7, ZmLTPd10, ZmLTPd11
Molecular Function	GO:0043177	organic acid binding	2.32E-09	1.53E-08	5	109	0.046	ZmLTPd3, ZmLTPd5, ZmLTPd7, ZmLTPd10, ZmLTPd11

Molecular Function	GO:0005488	binding	0.004856	0.026708	13	15770	0.001	ZmLTPg8, ZmLTPd3, ZmLTPd5, ZmLTPg12, ZmLTPd7, ZmLTP1.3-1, ZmLTP1.2-2, ZmLTPd10, ZmLTPd11, ZmLTPg25, ZmLTP1.7, ZmLTP1.8-1, ZmLTP1.8-2
Molecular Function	GO:0036094	small molecule binding	0.031808	0.137561	6	4812	0.001	ZmLTPd3, ZmLTPd5, ZmLTPd7, ZmLTPd10, ZmLTPd11, ZmLTP1.8-2
Molecular Function	GO:0043168	anion binding	0.033348	0.137561	6	4862	0.001	ZmLTPd3, ZmLTPd5, ZmLTPd7, ZmLTPd10, ZmLTPd11, ZmLTP1.8-2
Molecular Function	GO:0043167	ion binding	0.241639	0.886009	6	7922	0.001	ZmLTPd3, ZmLTPd5, ZmLTPd7, ZmLTPd10, ZmLTPd11, ZmLTP1.8-2
Molecular Function	GO:0004672	protein kinase activity	0.636801	1	1	1780	0.001	ZmLTP1.8-2
Molecular Function	GO:0016773	phosphotransferase activity, alcohol group as	0.679243	1	1	1989	0.001	ZmLTP1.8-2
Molecular Function	GO:0016491	oxidoreductase activity	0.682105	1	1	2004	0	ZmLTP1.7
Molecular Function	GO:0016301	kinase activity	0.762899	1	1	2489	0	ZmLTP1.8-2
Molecular Function	GO:0016772	transferase activity, transferring phosphorus-	0.808088	1	1	2832	0	ZmLTP1.8-2
Molecular Function	GO:0005524	ATP binding	0.893265	1	1	3755	0	ZmLTP1.8-2
Molecular Function	GO:0032559	adenyl ribonucleotide binding	0.896885	1	1	3808	0	ZmLTP1.8-2
Molecular Function	GO:0030554	adenyl nucleotide binding	0.898619	1	1	3834	0	ZmLTP1.8-2
Molecular Function	GO:0035639	purine ribonucleoside triphosphate binding	0.917761	1	1	4152	0	ZmLTP1.8-2
Molecular Function	GO:0001883	purine nucleoside binding	0.920606	1	1	4205	0	ZmLTP1.8-2
Molecular Function	GO:0032550	purine ribonucleoside binding	0.920606	1	1	4205	0	ZmLTP1.8-2
Molecular Function	GO:0032555	purine ribonucleotide binding	0.920606	1	1	4205	0	ZmLTP1.8-2
Molecular Function	GO:0001882	nucleoside binding	0.921238	1	1	4217	0	ZmLTP1.8-2
Molecular Function	GO:0032549	ribonucleoside binding	0.921238	1	1	4217	0	ZmLTP1.8-2
Molecular Function	GO:0017076	purine nucleotide binding	0.92228	1	1	4237	0	ZmLTP1.8-2
Molecular Function	GO:0032553	ribonucleotide binding	0.922899	1	1	4249	0	ZmLTP1.8-2

Molecular Function	GO:0097367	carbohydrate derivative binding	0.924477	1	1	4280	0	ZmLTP1.8-2
Molecular Function	GO:0000166	nucleotide binding	0.940949	1	1	4645	0	ZmLTP1.8-2
Molecular Function	GO:1901265	nucleoside phosphate binding	0.940949	1	1	4645	0	ZmLTP1.8-2
Molecular Function	GO:0016740	transferase activity	0.979903	1	1	6165	0	ZmLTP1.8-2
Molecular Function	GO:1901363	heterocyclic compound binding	0.999213	1	1	10044	0	ZmLTP1.8-2
Molecular Function	GO:0097159	organic cyclic compound binding	0.999228	1	1	10064	0	ZmLTP1.8-2
Molecular Function	GO:0003824	catalytic activity	0.999737	1	2	13695	0	ZmLTP1.7, ZmLTP1.8-2
Molecular Function	GO:0003674	molecular_function	1	1	13	23755	0.001	ZmLTPg8, ZmLTPd3, ZmLTPd5, ZmLTPg12, ZmLTPd7, ZmLTP1.3-1, ZmLTP1.2-2, ZmLTPd10, ZmLTPd11, ZmLTPg25, ZmLTP1.7, ZmLTP1.8-1, ZmLTP1.8-2

Note: Qvalue, corrected-pvalue; Fg_num, foreground gene number; Bg_num, background genenumber; Ratio= Fg_num / Bg_num.

Table S3. Primers for qPCR analysis of maize *LTP* genes

Primer	Forward (5'-3')	Reverse (5'-3')
q-anthr3	TCAGCATCATCAACAGCCTG	TGCCTTTTGCCCAGTGCATC
q-ms44	TGGGCATCATCAACAGCCTG	TGCCATCCATTGCAGCACGC
q-ZmLTP1.5	ACGTTCAGGTCCACGCTGTG	TGTTGTAGGACAGCGAGAGG
q-ZmLTP1.8-1	ATGCAGTAAAGGTTGTAAGG	TCTGAAAGCGTTATAGAGTGAG
q-ZmLTP2.9	TGCGATGTGACGCAGCTGAC	TCGTCCTTGTACCGGCACAG
q-ZmLTPd9	AGCTAGACCTGGACAAGGTG	TGCTCCATTCAGATTCAGCC
q-ZmLTPd15	AGATGATCCCCATGGCTGCAC	TGTCATGCAGTGCCTATCAC
q-ZmLTPd16	TGTCGTGAAGGTTGTGGATG	TGCACCCATCCCAGCAGTTG
q-ZmLTPg4	TCTTCGCCAACGCCTCCAAG	AGAAGAGTGACGAGGCGATC
q-ZmLTPg11	TCTACGCTTCCTGCGGAGG	ATAACAGGAACGGTGGCGGG
q-ZmLTPg14	ACTACGCGCTGTTCGCCAAC	AGCCTGGAGCCTGATAACGG
q-ZmLTPg15	AGCAGATGTTCAAGCTCCC	ACCATGATGGTGCTGCTGTG
q-ZmLTPg20	AGCCGACACAGCTGCAGATG	AGGCATACAGCAATGCGCAG
q-ZmLTPg26	ACTGCAGCATTGATCCTCGC	AGATCAAGATCGAGCGAGGC
q-ZmLTPx1	TCACCGTTGATGCCATCGC	AGCTGATGATGACGCACATG
q-ZmLTPx2	TCATCGCTTCCATCGACCTC	TAGAGCGCGAGGTGGTGG
ZmUbi2-RT	CGACAACGTGAAGGCGAAGA	ACGCAGATACCCAGGTACAGC
